# From clay pots to graphene membranes: the evolutionary journey of water purification media

**DOI:** 10.1039/d6ra05410b

**Published:** 2026-08-05

**Authors:** Arwa A. Makki, Dina Hajjar, Hatem M. Altass, Jan Mohamad Mir, Bashir Ahmad Malik, Saleh A. Ahmed

**Affiliations:** a Department of Biochemistry, College of Science, University of Jeddah Jeddah 80327 Saudi Arabia amaki@uj.edu.sa; b Department of Chemistry, Faculty of Science, Umm Al-Qura University 21955 Makkah Saudi Arabia saahmed@uqu.edu.sa saleh_63@hotmail.com; c Multidisciplinary Chemistry Laboratory, Department of Chemistry, Islamic University of Science and Technology India mirjanmohammad@gmail.com; d Computational Chemistry Laboratory, Department of PG Studies and Research in Chemistry and Pharmacy, RD University Jabalpur 482001 MP India

## Abstract

The quest for clean water is as ancient as civilization itself, evolving from the stochastic pores of clay vessels to the precisely engineered nanochannels of modern two-dimensional (2D) materials. Unlike previous reviews that focus primarily on material synthesis or specific applications, this review uniquely traces the conceptual lineage from ancient purification principles to modern 2D materials, demonstrating how atomic-scale engineering augments rather than replaces historical mechanisms. The survey provides a critical comparative framework that systematically evaluates performance across contaminant classes, identifies key research gaps, and proposes specific targets for practical implementation. This historical-to-horizontal perspective provides researchers with both contextual understanding and practical guidance. The study examines how carbon-based 2D platforms, graphene, graphene oxide (GO), reduced graphene oxide (rGO), and MXenes, have transformed water decontamination from a macroscale filtration art into an atomic-scale separation science. It reveals how these materials refine and augment historical purification mechanisms like size-exclusion sieving progresses from micron-scale randomness to ångström-level precision, adsorption shifts from general electrostatic capture to targeted chemical scavenging, and disinfection advances from leaching biocides to contact-based photothermal and catalytic destruction. Beyond inheriting ancient principles, 2D materials introduce novel functionalities including electrocatalytic degradation, plasmonic disinfection, and tunable ionic selectivity, enabling unprecedented removal of heavy metals, organic pollutants, pathogens, and emerging contaminants. However, despite remarkable laboratory performance, critical challenges in scalability, cost, stability, fouling, and environmental safety persist. This review not only deals with the state of the art but also provides a forward-looking framework for transitioning these advanced materials from bench-scale innovation to sustainable, real-world water treatment solutions.

## Abstract 2

The quest for clean water is as ancient as civilization itself, evolving from the stochastic pores of clay vessels to the precisely engineered nanochannels of modern two-dimensional (2D) materials.

## Graphical abstract

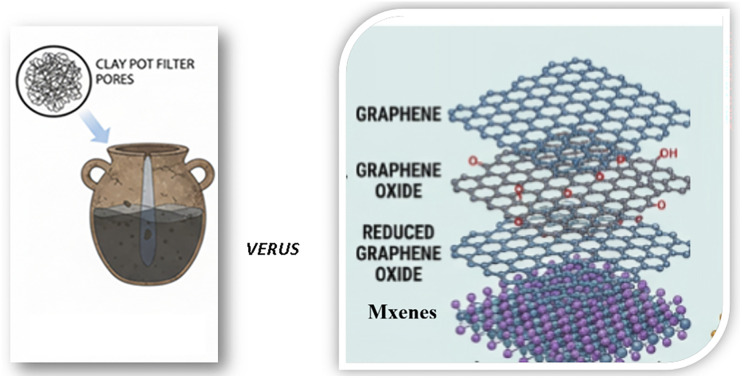

## 1. Introduction

Water purification is one of the most persistent technological challenges, evolving from ancient clay-based systems to modern membrane technologies.^1–3^ Globally, over 2.2 billion people lack access to safely managed drinking water, and water-related diseases cause more than 500 000 deaths annually (WHO, 2022).^4^ In the modern world, the global water crisis, driven by population growth, industrialization, and climate change, demands advanced, sustainable purification methods that overcome limitations of conventional approaches such as activated carbon adsorption, reverse osmosis, and chlorination.^5^ In this context, carbon-based two-dimensional materials have emerged from nanotechnology frontiers, offering revolutionary potential for water purification.^6–8^

The conceptual lineage from traditional porous media to engineered nanomaterials, demonstrating how carbon-based two-dimensional materials enhance fundamental decontamination mechanisms through atomic-scale precision, represents a fascinating area. Rather than representing a complete detachment from historical approaches, these materials emphasize the logical evolution and refinement of core purification principles first established in the ancient world. The fired clay vessel, a masterpiece of empirical materials engineering, synergistically combined size-exclusion sieving, adsorptive surface chemistry, and early forms of antimicrobial functionalization and bioremediation.^9–11^ Its stochastic, micron-scale pore network effectively removed pathogens and turbidity, establishing foundational paradigms of filtration.

However, the stand needs to be taken cautiously against suggesting a direct material lineage from clay to graphene. Instead, emphasizes intellectual continuity, the evolution of purification principles (sieving, adsorption, disinfection), and their implementation at progressively finer length scales. Where clay achieved separation through stochastic, micron-scale porosity, 2D materials achieve it through engineered, angstrom-scale channels.^12–14^ Where clay utilised passive cation exchange, 2D materials employ targeted chemical functionality and active catalysis.^15,16^ This is not a direct material progression but an intellectual refinement that results from the application of advancing scientific understanding to achieve the same purification goals with unprecedented precision and efficiency. The progress of human civilization from earthenware filters to contemporary ultrathin graphene oxide membranes represents continuous evolution marked by increasing control over material architecture. What clay accomplished randomly at micron scales, two-dimensional materials now achieve with atomic-scale deliberation.^17–20^ The transformation into angstrom-scale channels renders the ability to sieve individual ions. This advancement enables unprecedented control over water permeability and selectivity, moving beyond particulate and microbial contaminant removal to precise sieving of monovalent and divalent ions.

This review distinguishes itself from the numerous existing reviews on graphene-based materials for water purification^21–23^ through three innovations *viz*, an evolutionary framework that traces fundamental purification mechanisms from ancient clay to 2D materials, establishing continuity rather than presenting 2D materials as a revolutionary break followed by a critical assessment that systematically evaluates limitations including scalability, cost, stability, fouling, and environmental safety with quantitative benchmarking. Finally, an application-oriented roadmap proposes specific targets for material design, synthesis scale-up, and system integration. This historical-critical-applied approach provides researchers with both fundamental understanding and actionable guidance. In the context of water purification agents, this review details the evolutionary journey, systematically examining how materials such as graphene, graphene oxide, and MXenes have surpassed historical precursors, addressing long-standing limitations in flow rate, chemical resistance, and specificity that have challenged traditional porous media.^24–26^ The study involves a critical examination of this technological evolution through three analytical lenses, namely, mechanistic comparison between traditional and advanced materials, performance evaluation across contaminant classes, and scalability assessment for practical implementation. Also, the systematic analysis of how graphene, graphene oxide, reduced graphene oxide, and MXenes overcome the limitations of conventional materials has been addressed. Meanwhile, introducing novel functionalities through their unique electronic, mechanical, and chemical properties is also the main part of the discussion. Particular emphasis is placed on structure–property relationships that govern decontamination efficiency, with critical evaluation of synthesis–property–performance correlations. The subsequent sections are organized first to establish historical context, then examine 2D material fundamentals, followed by critical performance analysis across applications, and concluding with identified research gaps and future directions. This structured approach enables both comprehensive coverage and analytical depth, providing researchers with not only a summary of current knowledge but also a critical framework for advancing the field toward sustainable water treatment solutions.

## 2. Bridging eras: from clay porous matrices to atomic-scale two-dimensional materials

The fundamental human requirement for potable water has driven technological innovation since ancient civilizations.^27–29^ Among the most universal historical solutions was the utilization of clay, a naturally abundant and amenable material employed across diverse cultures from the Indus Valley to Nile River societies.^30–32^ These civilizations independently developed water storage in fired ceramic vessels that functioned as passive purifying systems requiring no external energy beyond initial fabrication. Their effectiveness derived from synergistic physicochemical mechanisms rather than single processes.

The primary decontamination pathway was physical size exclusion, microfiltration through complex, targeting pore networks typically ranging from 0.1 to 10 micrometers in diameter.^33–35^ This porous architecture physically strains suspended particulates, turbidity, and larger pathogenic organisms, including common waterborne bacteria and protozoan cysts.^36–38^ However, viruses (20–100 nm) are smaller than the typical clay pore size and can pass through unless captured by adsorption. Beyond simple sieving, clay surfaces provide chemically active substrates for contaminant immobilization. The crystalline structure of clay minerals like montmorillonite imparts a negative surface charge, facilitating cation exchange capacity that effectively sequesters dissolved heavy metal species, including lead and copper.^39–41^ Even some viruses, smaller than pore sizes, could be removed through electrostatic surface interactions. Over time, beneficial microbial biofilms colonized vessel interiors, functioning as living filters that biologically degrade organic pollutants and outcompete harmful bacteria, an early application of bioremediation.^42,43^

To provide a scientifically rigorous foundation for the clay-based mechanisms, the chemical detail refers clay minerals as phyllosilicates with a layered structure; common species include kaolinite (Al_2_Si_2_O_5_(OH)_4_), montmorillonite ((Na, Ca)_0_._33_(Al, Mg)_2_(Si_4_O_10_)(OH)_2_·nH_2_O), and illite (K_0_._5_(Al, Mg)_2_(Si_3_._5_Al_0_._5_)O_10_(OH)_2_). Their permanent negative charge arises from isomorphous substitution (*e.g.*, Al^3+^ replacing Si^4+^, Mg^2+^ replacing Al^3+^), leading to cation exchange capacities (CEC) typically in the range of 10–150 meq/100 g. The dominant heavy-metal removal mechanism is cation exchange:Clay^−^Na^+^ + M^2+^(aq.) ⇌ ≡Clay^−^M^2+^ + 2Na^+^(aq.)

**Fig. 1:**
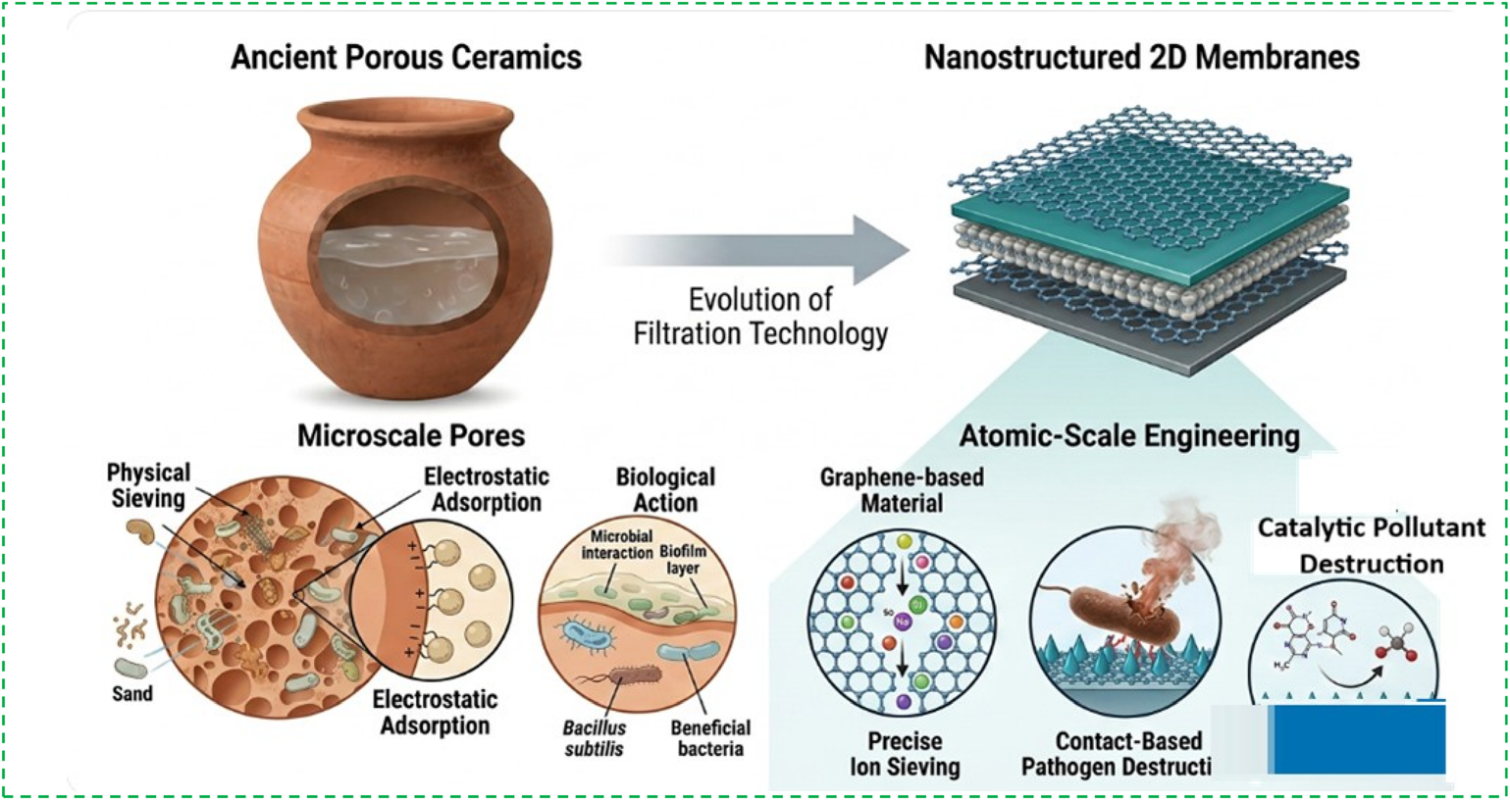
The conceptual evolution of water purification mechanisms: ancient clay vessels (left) *versus* modern 2D material membranes (right).

Selectivity follows the order Pb^2+^ > Cu^2+^ > Cd^2+^ > Zn^2+^ > Ni^2+^, and is strongly pH-dependent because edge sites (≡Si–OH, ≡Al–OH_2_^+^) protonate/deprotonate, affecting metal binding. Additionally, biofilm extracellular polymeric substances (EPS) contain carboxyl, phosphoryl, and hydroxyl groups that complex metals and enhance biosorption.^44–46^ While clay systems established foundational purification principles, their stochastic pore architecture, limited adsorption specificity, and inconsistent performance represent inherent limitations. In the course of human civilization advancements, the intentional mixing of clay with organic temper (rice husks, sawdust) constituted early materials engineering to control porosity, and the silver lining of vessels represented pioneering antimicrobial functionalization.^46,47^ These practices established core concepts that modern materials science has refined through atomic-scale engineering rather than replaced entirely (Fig. 1).

## 3. Material evolution: from empirical processing to controlled synthesis

Clay vessel performance is linked with the results recorded from generations of refined empirical knowledge in materials processing. Artisans systematically manipulated clay composition by intentionally mixing it with specific organic temper materials whose type, grain size, and proportion were critical design variables controlling final microstructure.^48,49^ During kiln-firing (typically 800–900 °C), combustible temper particles burned away, creating deliberate micropore networks that dictated filter flow rates and pathogen-removal efficacy. This practice represents foundational materials engineering, where processing parameters were optimized through observed outcomes before the underlying science was formally understood.

Therefore, the core idea was the functionalization of clay surfaces with antimicrobial agents, most notably elemental silver.^50–52^ Cultures worldwide observed improved water quality in silver-lined containers or jars containing silver coins, noting extended storage longevity and reduced microbial growth. In modern materials science, this practice has been classified as pioneering antimicrobial surface functionalization, where steady silver ion (Ag^+^) release through oxidative dissolution creates low-concentration colloidal suspensions that disrupt bacterial cell membranes and interfere with metabolic enzymes.^53–55^ This feature of vessels from purely physico-chemical filters into composite chemical–biological systems, providing continuous, low-level disinfection that addresses pathogens small enough to pass through physical pores.

The limitations of clay vessels, including large irregular pore sizes, low flow rates, and inability to remove dissolved salts or small-molecule contaminants, stimulated the search for more advanced materials.^56–59^ While effective for millennia, clay filters possess stochastic pore networks primarily in the micrometer range, ineffective for dissolved ionic species and small-molecule organic pollutants. Indirect water pathways result in inherently low hydraulic flow rates, making large-volume processing impractical. Mechanical fragility presents additional deployment challenges. Therefore, these drawbacks drove scientific inquiry toward substances with precisely defined nanostructures.

**Fig. 2:**
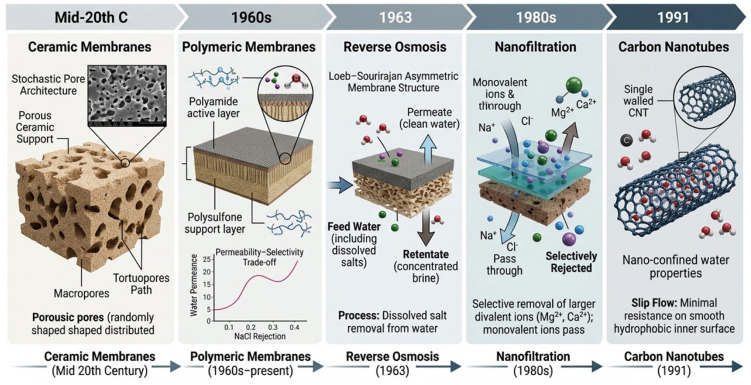
Historical progression of membrane technologies towards 2D materials.

Rather than a direct leap from historical clay-based media to contemporary 2D materials, the evolution of membrane science progressed through several foundational intermediate technologies that collectively established the requisite physicochemical and engineering principles (Fig. 2). Beginning in the mid-20th century, ceramic membranes offered enhanced mechanical robustness through porous supports, yet their inherently stochastic pore architectures precluded precise molecular discrimination.^60^ The subsequent proliferation of polymeric membranes from the 1960s onward, exemplified by polyamide and polysulfone materials, introduced tailored selective permeability, though their utility remained fundamentally circumscribed by the ubiquitous permeability–selectivity trade-off.^61^ Building upon this, the Loeb–Sourirajan reverse osmosis process in 1963 enabled the effective removal of dissolved salts, albeit at the cost of high hydraulic pressures and substantial energy demands, while the advent of nanofiltration in the 1980s refined this capability by achieving selective retention of divalent ions alongside permeation of monovalent species.^62^ A pivotal mechanistic breakthrough arrived with Iijima's discovery of carbon nanotubes in 1991,^63,64^ which demonstrated that atomically smooth, hydrophobic carbon surfaces promote pronounced slip flow, yielding water transport rates two to four orders of magnitude greater than classical Poiseuille flow predictions, and further revealed that water confined within nanoscale conduits exhibits thermophysical properties distinctly different from those of the bulk phase. Crucially, these nanotube-derived insights into frictionless interfacial transport and nanoconfinement effects directly furnished the scientific framework, both conceptual and experimental, that later enabled the interpretation, prediction, and engineering of fluid and ion transport through the emergent class of 2D material membranes. Thus, 2D materials represent the current frontier, offering atomic-scale control over channel dimensions and surface chemistry, but they build upon decades of membrane transport theory and materials engineering.^65,66^

The paradigm shift in materials science occurred with the isolation of graphene by Geim and Novoselov,^67^ demonstrating that perfectly two-dimensional, atomically thin crystals are stable under ambient conditions. Graphene's hexagonal carbon lattice became the first true two-dimensional material, inaugurating research dedicated to atomically thin solids.^68,69^ This represented a radical departure from conventional three-dimensional material paradigms, with unique electron confinement in two dimensions granting extraordinary properties: unparalleled mechanical strength, exceptional electrical and thermal conductivity, and immense theoretical specific surface area.

A crucial link in the narrative is activated carbon, which is the most widely used adsorbent in water treatment (global annual consumption >1 million tons).^70,71^ Its microporous structure (0.5–5 nm) provides specific surface areas of 500–1500 m^2^ g^−1^ and surface functional groups (carboxyl, phenolic, lactone, carbonyl) that confer moderate adsorption capacity for polar contaminants through hydrogen bonding and electrostatic interactions.^72,73^ However, compared to GO, activated carbon offers lower specific interaction sites, poorer molecular selectivity, and requires larger dosages (typically 5–25 g L^−1^*versus* 0.1–1 g L^−1^ for GO-based adsorbents). The transition from amorphous carbon materials to 2D graphene derivatives represents a fundamental paradigm shift from predominantly pore-limited diffusion control to surface-dominated adsorption with engineered chemical functionality.^74–76^ This shift is analogous to moving from a sponge (random pores) to a well-ordered molecular sieve.

For water purification, the most critical attribute is the ability to create membranes with controlled, angstrom-scale dimensions. Unlike irregular clay voids, gaps between two-dimensional nanosheets can be engineered with sub-nanometer precision, enabling molecular-level sieving based on hydrated ionic radii. The quest for advanced filters thus evolved from relying on naturally occurring porosity to actively engineering it at atomic scales. The discovery of graphene enabled exploration of an extensive two-dimensional materials family including graphene oxide, transition metal dichalcogenides, and MXenes,^77–79^ each offering distinct chemical functionality, electronic properties, and surface characteristics. Researchers can now design layered heterostructures combining different two-dimensional layers, creating smart membranes with tailored selectivity for specific contaminants.

The revolution lies not merely in the materials themselves but in the newfound ability to control material architecture across length scales from the atomic upward, transitioning from passive filtration to active, selective separation. The field has progressed from utilizing naturally available materials to constructing *de novo* nanoscale architectures optimized for specific separation tasks. Therefore, two-dimensional materials represent the culmination of this evolutionary journey, offering platforms to overcome fundamental physicochemical limitations that constrained traditional porous media for centuries.

## 4. The two-dimensional materials family: structure, properties, and functionality

**Fig. 3:**
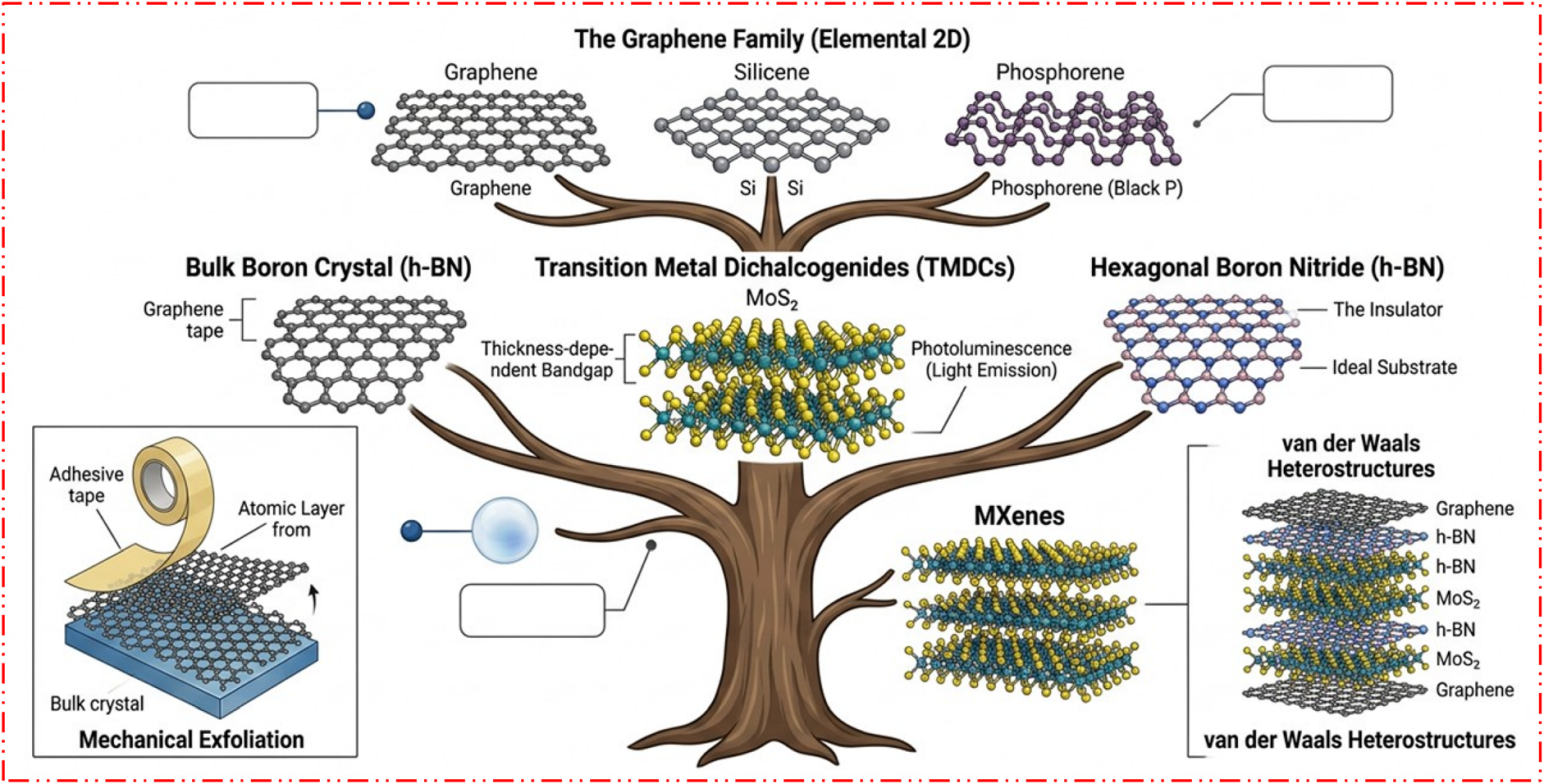
Illustration of the family tree of key 2D materials used in water decontamination.

Two-dimensional materials exhibit profound structural anisotropy (Fig. 3), extending over micrometers in two dimensions while remaining near-atomic in thickness in the third. Typically isolated through mechanical or chemical exfoliation of van der Waals-bonded bulk precursors, this unique geometry liberates them from substrate dependence common to conventional thin films, granting unprecedented structural and functional independence.^80,81^

### 4.1. Ultra-high aspect ratio and surface area

A single gram of graphene theoretically provides over 2600 m^2^ of surface area, creating an immense platform for contaminant interaction.^82,83^ This extraordinary surface-to-mass ratio enables highly efficient adsorption with minimal material requirements. However, in practice, aggregation in aqueous environments reduces effective surface area by 30–70%. For GO and rGO, measured surface areas typically range from 400–1500 m^2^ g^−1^, depending on synthesis and processing. Unlike bulky filter materials where only exterior surfaces are active, graphene's entire structure functions as an interactive surface working at maximum efficiency. For water purification, this means heavy metal ions or organic dyes are swiftly and effectively adsorbed onto this extensive carbon canvas (Table 1). The high surface-to-mass ratio enables lightweight, portable, highly effective personal water filters or air purifiers. Interactions can be physical (contaminants trapped in nanoscale wrinkles and folds) or chemical (tailored surfaces specifically attracting certain contaminants). This versatility makes graphene platforms adaptable for diverse targets, including oil spills, industrial waste, or pharmaceutical residues.

**Table 1:** Comparative properties of 2D materials for water treatment

Material	Surface area (m^2^ g^−1^)	Electrical conductivity (S m^−1^)	Mechanical strength (GPa)	Key functional groups	Primary mechanisms	Critical limitations
Graphene	2630 (theoretical) 500–1500 (practical)	10^6^	1300	None (pristine)	Sieving, conduction	Poor dispersibility, difficult processing, high cost
GO	400–800 (measured)	10^−4^–10^2^	200–500	–OH, –COOH, –O–	Adsorption, sieving	Swelling in water, pH sensitivity, enzymatic degradation
rGO	500–1500	10^2^–10^5^	400–800	Residual O-groups	Adsorption, catalysis	Incomplete reduction, lower adsorption capacity than GO
MXenes	10–200	10^4^–10^5^	300–500	–O, –OH, –F	Capacitance, catalysis	HF-based synthesis, oxidation in water, high cost

Each material class offers distinct advantages and limitations. While graphene provides unparalleled conductivity and strength, its hydrophobicity and processing challenges limit aqueous applications. GO offers superior dispersibility and adsorption capacity but suffers from reduced conductivity and stability. rGO balances functionality and conductivity but requires careful reduction control. MXenes provide unique metallic conductivity and surface functionality but face synthesis scalability challenges and oxidation instability. Optimal material selection depends on target contaminants and operational conditions.

### 4.2. Tunable chemical functionality

The surfaces of two-dimensional materials can be chemically modified with various functional groups, allowing precise engineering of interactions with specific pollutants.^84,85^ This chemical programmability enables materials to selectively target single pollutant types from complex mixtures. Graphene oxide comes pre-equipped with oxygen-containing groups (epoxides, hydroxyls) that act as charged docking stations, transforming material personality and affinity for different contaminants. By carefully selecting chemical attachments, researchers can engineer materials with predetermined targets. For heavy metal capture like lead or mercury, surfaces can be tailored with sulfur-containing groups forming strong bonds with toxic ions, creating specialized hunters that ignore harmless minerals while selectively sequestering dangerous metals. For organic dye removal from industrial wastewater, surfaces can be modified to increase or decrease hydrophobicity to attract specific molecules. This chemical engineering enables intelligent, selective extraction rather than simple filtration.

### 4.3. Exceptional mechanical strength and flexibility

Graphene is the strongest material ever measured, allowing the creation of ultrathin, robust, flexible membranes.^86,87^ This exceptional mechanical strength forms the foundation for durability in water treatment systems. Robust filter materials withstand immense water pressures without fracturing, ensuring long-term structural integrity. Ultrathin graphene oxide membranes resist tearing or rupture under constant water flow stress. Unlike brittle ceramic filters, graphene-based filters can flex, bend, or twist during handling and installation without damage. This strength–flexibility combination enables freestanding, paper-like filters that won't disintegrate in water. Durability translates directly to reusability; robust filters undergo multiple cleaning cycles (backwashing, chemical washing) without degradation. In dynamic water environments, innate flexibility allows absorption of hydraulic shocks and pressure surges without failure. Atomic thinness coupled with high tensile strength enables membranes that are both highly selective and remarkably tough.

### 4.4. Unique electronic and optical properties

Properties high electrical conductivity and plasmonic effects enable novel purification mechanisms, including electrocatalytic degradation and photothermal disinfection.^88–90^ The high conductivity character transforms passive filters into active, electrified water treatment reactors. Submerged graphene-based electrodes serve as microscopic stages for electrocuting pollutants, breaking them down at the molecular level. This electrocatalytic degradation targets stubborn organic pollutants resistant to conventional filtering methods.

When current is applied, conductive graphene surfaces facilitate chemical reactions, generating powerful oxidants like hydroxyl radicals directly at contamination sites.^91,92^ These radicals function as molecular scalpels, surgically dismantling complex toxic compounds into harmless water and carbon dioxide. Meanwhile, MXenes introduce light-harnessing capabilities for instant heat generation. MXenes exhibit strong plasmonic effects where electrons oscillate collectively under specific light wavelengths, converting light energy into intense localized heat with astonishing efficiency.

### 4.5. Critical limitations of 2D materials

A balanced assessment of 2D materials must move beyond their remarkable properties to confront critical limitations that currently hinder their practical application.^93,94^ The most fundamental challenge is stability, as materials like GO are prone to reduction under sunlight or heat, compromising their hydrophilicity, while MXenes degrade through oxidation in aerated water, and both can be subject to enzymatic breakdown in biological environments. This inherent instability is compounded by the issue of swelling; upon hydration, GO membranes can expand their interlayer spacing from approximately 0.6 nm to over 1.2 nm, drastically reducing ion rejection capabilities from over 95% to as low as 60–70%.^95,96^ Even if stability and swelling are managed, operational performance is severely threatened by fouling, where organic matter, biofilms, and particulates can diminish membrane flux by 30–60% within just 24–72 hours of exposure to real wastewater. The scale-up challenge is inseparable from these performance issues; transitioning from laboratory-scale membranes of 1–100 cm^2^ to industrial modules requiring 1–10 m^2^ is fraught with difficulty, as even a 0.1% defect density can halve selectivity, and commercial roll-to-roll production at viable speeds is not yet established. Economically, the path to industrialization is blocked by prohibitive material costs, GO is priced at $100–500 per g, a staggering >10 000-fold increase over activated carbon, making it impractical for large-scale municipal use, while MXenes are even more expensive at over $1000 per g. Finally, the environmental footprint cannot be overlooked, as GO exhibits acute toxicity to aquatic organisms like *Daphnia magna* at concentrations of 10–50 mg L^−1^ and chronic sublethal effects at lower levels, with the release of these nanomaterials during operation and disposal posing significant, and for MXenes largely unstudied, ecological risks.^97,98^ Together, these limitations, spanning stability, swelling, fouling, scalability, cost, and toxicity, constitute a formidable barrier that must be overcome before 2D materials can transition from scientific curiosity to practical technology.

## 5. Carbon-based two-dimensional materials: a spectrum of possibilities

Among two-dimensional materials, carbon-based structures are particularly prominent in environmental applications due to their biocompatibility, high conductivity, and excellent mechanical strength.^99–101^ This unique combination, structural integrity, outstanding electrical performance, and natural biological affinity, makes carbon two-dimensional materials uniquely suited for ecological applications.

### 5.1. Graphene: the fundamental building block

**Fig. 4:**
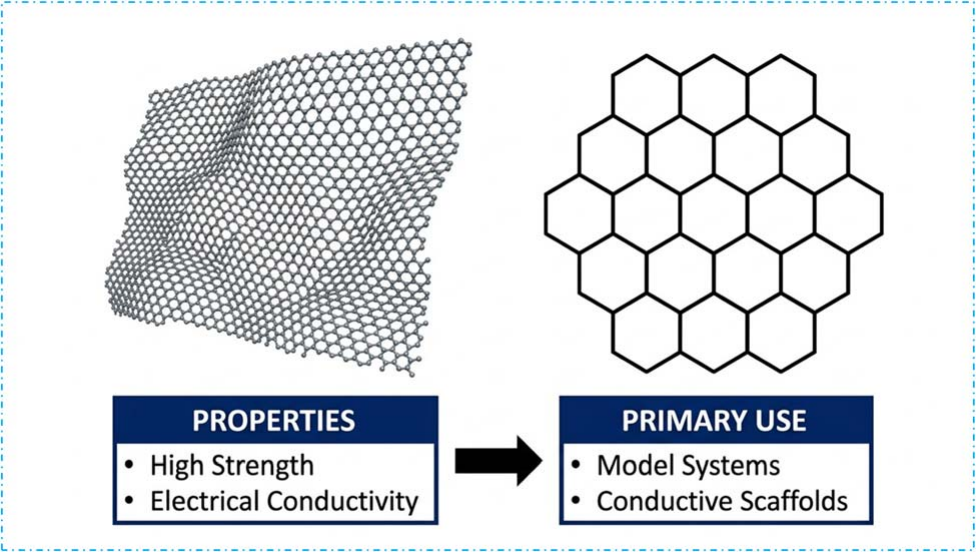
Graphene and its properties.

As illustrated in Fig. 4, graphene serves as the ultimate foundational unit with a single layer of sp^2^-hybridized carbon atoms in a perfect hexagonal lattice. This bonding creates a flawlessly flat, two-dimensional canvas one atom thick. Its pristine, defect-free surface offers an unparalleled landscape devoid of three-dimensional material irregularities. This atomic smoothness is critical for investigating fundamental physics, eliminating scattering sites that obscure intrinsic electron behavior. The perfectly ordered hexagonal lattice acts as an ideal model system for probing quantum transport and exotic phenomena in confined dimensions. However, hydrophobicity and lack of functional groups in pristine graphene limit its direct use in aqueous adsorption. Most water-treatment applications use derivatives (GO, rGO) with oxygen functionalities.^102–104^

### 5.2. Graphene oxide: chemical versatility

**Fig. 5:**
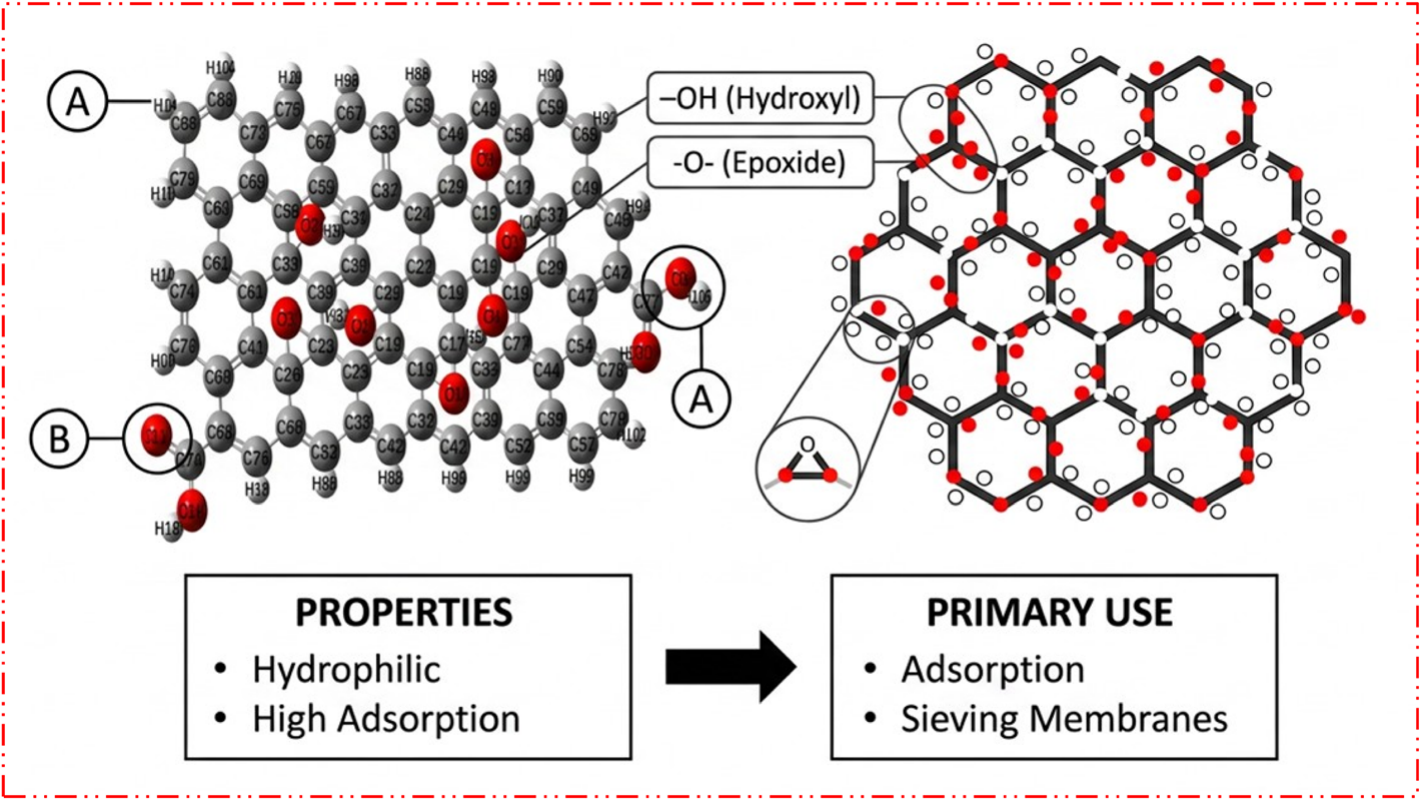
Graphene oxide (GO): (icon: hexagonal lattice with red/white O/H groups) and its properties.

Graphene oxide is graphene decorated with oxygen-containing functional groups (hydroxyl, epoxy, carboxyl),^105–107^ (Fig. 5). This chemical decoration fundamentally transforms inherent graphene properties. Where pristine graphene is hydrophobic, polar functional groups render graphene oxide intensely hydrophilic, giving it innate water affinity. This high hydrophilicity allows graphene oxide sheets to disperse readily in aqueous solutions, forming stable colloidal suspensions crucial for solution-based processing. The oxygenated graphene oxide landscape acts as a dense molecular docking network providing abundant, highly active adsorption sites. Positively charged metal ions in water (lead, cadmium, copper) are powerfully attracted to and captured by negatively charged oxygen sites.^108^ Beyond heavy metals, polar functional groups enable strong interactions with diverse polar organic molecules, including dyes and pharmaceuticals. Epoxy and hydroxyl groups positioned on sheet surfaces create disrupted but highly accessible planes for contaminant binding. Carboxyl groups at sheet edges are particularly reactive, often serving as primary anchors coordinating strongly with metal cations. The adsorption capacity of GO for Pb^2+^ can reach 842–1200 mg g^−1^ under optimized lab conditions (pH 5–6, no competing ions), but this drops to 200–500 mg g^−1^ in realistic wastewater due to competing cations (Ca^2+^, Mg^2+^) and natural organic matter.^109,110^

### 5.3. Reduced graphene oxide: the versatile compromise

**Fig. 6:**
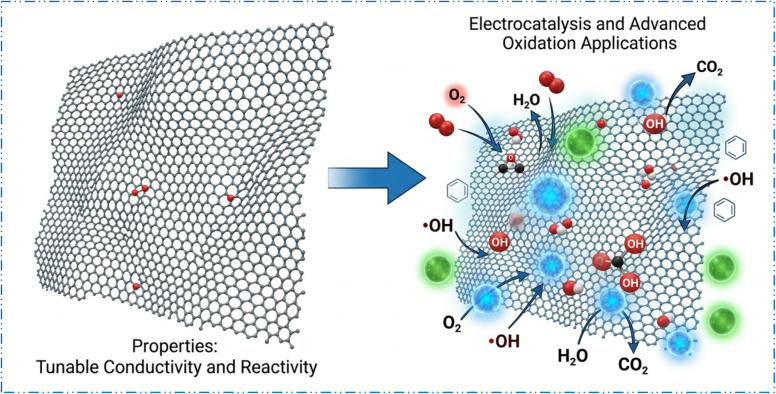
Reduced GO (rGO): (icon: a hybrid of graphene and GO) and its properties.

Reduced graphene oxide, as illustrated in Fig. 6, occupies an important middle ground in the two-dimensional materials family.^111–113^ This chemically tailored graphene oxide undergoes reduction processes, partially restoring lost conductivity while intentionally retaining strategic oxygen group residues on surfaces and edges. This creates a hybrid material capturing the best of both worlds: some conductivity and some reactivity. Reduced graphene oxide is not a failed pure graphene attempt but a deliberately engineered material with distinct, valuable property sets. rGO offers better stability against swelling than GO, but its adsorption capacity for heavy metals is typically 30–50% lower than GO's because of the reduced number of oxygen groups. It is particularly suited for photocatalytic and electrocatalytic applications where conductivity is essential.

### 5.4. MXenes and other 2-dimensional catalysts

**Fig. 7:**
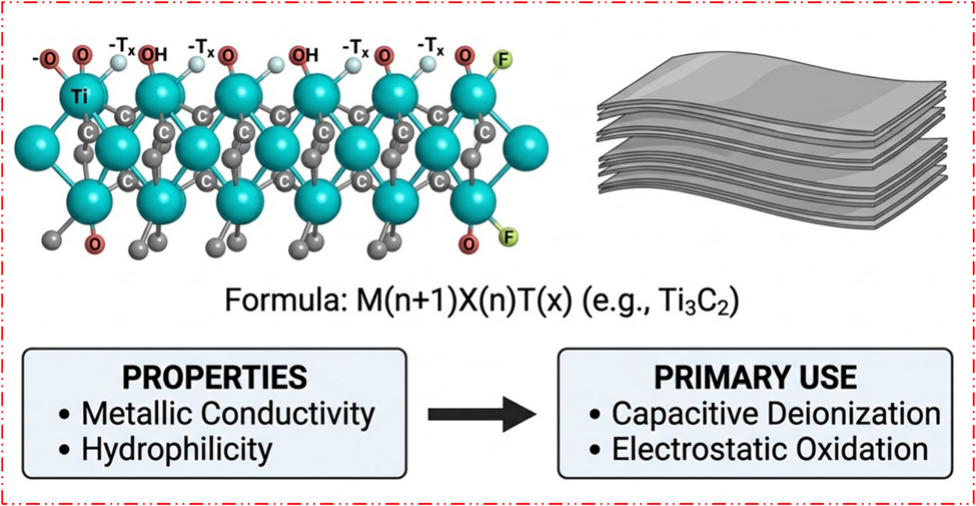
2D-materials having iconic formula M_*n*+1_X_*n*_T_*x*_, *e.g.*, Ti_3_C_2_ and properties.

Beyond carbon-centric graphene, MXenes have emerged as veritable materials science powerhouses.^114–116^ These complex compounds, two-dimensional transition metal carbides, nitrides, and carbonitrides, are of the formula M_*n*+1_X_*n*_T_*x*_. M represents early transition metals (titanium, molybdenum), providing robust metallic backbones (Fig. 7). X sites are occupied by carbon and/or nitrogen atoms nestled between metal atoms. Crucially, T_*x*_ symbolizes vibrant surface functional group coats (oxygen, hydroxyl, fluorine) acquired during synthesis. This surface termination dictates material chemical behavior and reactivity.

A defining MXene trait is innate metallic conductivity, allowing electrons to travel freely across two-dimensional planes. Unlike many conductive materials, MXenes are also inherently hydrophilic with natural water affinity and ready dispersion. This rare, highly conductive, and water-dispersible combination makes them exceptionally suited for aqueous electrochemistry. In electrocatalysis, MXenes provide ideal conductive platforms hosting active sites driving crucial reactions like hydrogen evolution. Low electrical resistance ensures minimal energy loss, making processes like oxygen reduction for fuel cells highly efficient. For water purification, MXene conductivity is harnessed in capacitive deionization, an effective, energy-efficient desalination technology.^117–119^ In capacitive deionisation, MXene electrodes act as electrical sponges; when charged, they electrostatically attract and sequester salt ions directly from water. Rich surface chemistry provided by oxygen and hydroxyl groups creates abundant heavy metal ion and polar organic contaminant adsorption sites. However, MXenes suffer from oxidation in aqueous environments, particularly in the presence of dissolved oxygen. For example, Ti_3_C_2_T_*x*_ loses 20–30% of its capacitance after 50 cycles in water. Surface coatings and antioxidant additives are being explored to mitigate this.^120–122^

Table 2 summarises critical comparative properties. Graphene and MXenes offer superior conductivity but suffer from high costs and poor scalability/stability, while GO provides excellent dispersibility and adsorption at the cost of poor conductivity and membrane stability. rGO emerges as the most balanced compromise, delivering moderate-to-good performance across all criteria, including conductivity, reusability, and scalability, without the extreme weaknesses of its counterparts. No single material universally excels; optimal selection depends entirely on application priorities, GO for adsorption-based water treatment, graphene/MXenes for electronic or high-conductivity uses, and rGO for integrated systems requiring a middle-ground performance profile.^123–125^

**Table 2:** Comparative critical properties of the materials

Criteria	Graphene	GO	rGO	MXenes
Synthesis cost	Very high	Moderate	Moderate	Very high
Water dispersibility	Poor	Excellent	Good	Good
Surface area (practical)	500–1500	400–800	500–1500	10–200
Adsorption capacity	Low	High	Moderate	Moderate–high
Selectivity control	Limited	Good	Moderate	Good
Conductivity	Excellent	Poor	Good	Excellent
Membrane stability	Good	Poor (swelling)	Moderate	Poor (oxidation)
Scalability	Poor	Moderate	Moderate	Very poor
Reusability	Good	Moderate	Good	Limited

For targeted water treatment applications, the distinct physicochemical properties of each material dictate clear performance leaders. Heavy metal removal is best served by GO, owing to its abundant oxygenated functional groups and high adsorption capacity, while organic dye removal benefits from the π–π stacking and electrostatic interactions offered by both GO and rGO. Desalination applications favor graphene or rGO due to their superior selectivity and water permeability. For photocatalytic degradation, the synergistic conductivity and light-response capabilities of rGO and MXenes make them the optimal choice, whereas capacitive deionization uniquely exploits high electrical conductivity and ion-intercalation properties of MXenes. Lastly, antimicrobial filtration leverages the physical sharp edges and oxidative stress mechanisms of GO and MXenes, underscoring that no single material is universally superior, rather, the selection must be strategically tailored to the specific contaminant and operational mechanism of each treatment process.^126–128^

## 6. A direct comparison: mechanistic evolution from clay to two-dimensional materials

The progression from clay to two-dimensional materials represents refinement and augmentation of core purification principles rather than replacement. Fundamental water purification principles, sieving, adsorption, and disinfection, have remained remarkably constant across millennia. The shift from conventional clay vessels to engineered two-dimensional materials like graphene oxide and reduced graphene oxide is not the abandonment of ancient principles but their ultimate atomic-scale augmentation. This progression mirrors transportation evolution from wheels to modern automobiles; core movement functions remain while mechanisms transform through precision, control, and power.

### 6.1. Historical purification mechanisms (ancient to pre-industrial)

Clay vessels function primarily through macro- and micro-pore networks (0.1–10 µm), effectively filtering larger pathogens like bacteria and protozoa through size-exclusion.^129–131^ However, this porosity is inherently stochastic, consisting of irregular, tortuous pathways that impose significant flow rate limitations and offer minimal dissolved contaminant selectivity. Chemical mechanisms include cation exchange (CEC 10–150 meq/100 g), surface complexation at edge sites, and biosorption by biofilm EPS. Removal efficiencies of bacteria 70–95%, protozoa >99%, viruses 50–90% (pH-dependent), heavy metals 10–80% depending on ion and conditions.

### 6.2. Modern purification mechanisms (20th century)

The 20th century introduced activated carbon (surface area 500–1500 m^2^ g^−1^, capacity 100 mg g^−1^ Pb^2+^), polymeric and ceramic membranes (microfiltration, ultrafiltration, reverse osmosis), and chemical disinfection (chlorine, ozone). These technologies improved efficiency but still suffered from trade-offs between permeability and selectivity, high energy consumption, and disinfection by-products.^132–134^

### 6.3. 2D material purification mechanisms (21st century)

In stark contrast, carbon-based two-dimensional materials have mastered sieving with near-atomic precision. Carefully stacking graphene oxide sheets creates nano- and ångström-scale channels with defined interlayer spacing, enabling feats impossible for clay,^135–137^ such as discriminating between hydrated ions, filtering salt, or separating monovalent from divalent cations. Chaotic clay flow is replaced by highly ordered, laminar flow channels, maximising efficiency and selectivity unattainable naturally. This represents the first great evolutionary advance: transition from stochastic porosity to engineered molecular sieves. In adsorption, an atom or a molecule adheres to surfaces, representing another connecting thread across technological eras. Clay possesses natural, albeit limited, adsorption capacity primarily through cation exchange due to inherent mineral constituent negative charges, moderately effective for positive metal ions but largely ineffective for neutral organics or anions. Two-dimensional materials have transformed adsorption into a high-capacity, highly specific science. With theoretical surface areas exceeding 2600 m^2^ g^−1^ for graphene, physical interaction platforms are astronomically larger than what is reported in clay. More importantly, materials like graphene oxide feature tunable rather than fixed surface chemistry. Oxygen-containing functional groups on graphene oxide provide abundant, specific heavy metal ion complexation sites. These surfaces can be further functionalised with organic molecules targeting specific pesticides or pharmaceuticals, creating designer adsorbents. The evolutionary advance here is movement from passive, generalist adsorption to active, targeted, high-capacity scavenging processes.

### 6.4. Adsorption: from general to specific interactions

Clay adsorption relies primarily on cation exchange capacity (CEC ≈ 10–150 meq/100 g) through electrostatic interactions. 2D materials offer vastly superior adsorption through multiple mechanisms, namely coordination with oxygen groups on GO (200–1500 mg g^−1^ for heavy metals), π–π stacking on graphene/rGO surfaces for organics, and electrostatic/chemical interactions on MXenes. While adsorption capacities are impressive, most studies report idealized conditions (single contaminants, optimized pH). Real wastewater contains competing ions and complex matrices that significantly reduce performance, a critical gap requiring systematic investigation. For example, Cd^2+^ adsorption on GO decreases by 40–60% in the presence of Ca^2+^/Mg^2+^, and Pb^2+^ uptake can drop by 50% in humic acid-containing waters.

### 6.5. Antimicrobial action

Historical clay shows antimicrobial action relied on elegant but crude mechanisms, including antimicrobial ion leaching, most commonly silver. While broad-spectrum and effective, this is consumptive; biocides leach out, deplete over time, and can enter environments and food chains, potentially fostering resistance. On the other hand, two-dimensional materials offer more sophisticated, sustainable antimicrobial tactics. One mechanism is direct physical damage where sharp graphene oxide or MXene sheet edges act as nano-knives, surgically slicing bacterial cell membranes upon contact.^138–140^ Another is oxidative stress generation, where materials catalyze reactive oxygen species production that oxidize and destroy cellular components. Furthermore, materials like MXenes exhibit powerful photothermal properties, converting light into intense local heat, literally cooking pathogens without chemical release. This multi-modal, contact-based approach marks a monumental advance, moving from depleting, leach-based strategies to renewable, physical, and catalytic methods, minimizing environmental impact. Comparative studies show MXenes achieve 5-log *E. coli* reduction in 2 hours (at 200 µg mL^−1^, shaking conditions), GO requires 4 hours, and Ag nanoparticles require 6 hours. However, chlorine achieves >6-log reduction in 30 minutes, highlighting that 2D materials are not yet competitive for rapid disinfection but offer distinct advantages in eliminating chemical additives and minimizing toxic by-product formation.^141–147^

### 6.6. Catalytic degradation

Pertinent to this, the most significant augmentation is introducing entirely new purification mechanisms, *e.g.*, catalytic degradation. Traditional clay pot technology lacked this feature; it is a capability born from unique two-dimensional material electronic properties. Materials like reduced graphene oxide with restored conductivity or certain MXenes can act as powerful electrocatalysts. They can activate stable oxidants like peroxymonosulfate or generate hydroxyl radicals *in situ* to attack and mineralize persistent organic pollutants. These are not merely filtered out, but they are broken down into benign components (water, carbon dioxide), solving contaminant concentration and disposal problems. This represents ultimate augmentation that is moving beyond containment to destruction. Mechanistically, rGO-PMS systems follow both radical pathways (SO_4_˙^−^, ˙OH) and non-radical pathways (singlet oxygen ^1^O_2_, electron transfer), defect density and oxygen groups play critical roles. For instance, N-doped rGO shows 3× higher PMS activation than pristine rGO.^148–150^

### 6.7. Quantitative performance comparison

In conclusion, the lineage from clay to graphene, the stochastic sieve refinement into a precision instrument for ion selection,^151–153^ is quite fascinating. As highlighted in Table 3, the evolutionary advancement from clay to 2D materials can be summarised as a progression from stochastic to engineered, passive to active, general to specific, and containment to destruction. This is not merely incremental improvement but a paradigm shift enabled by atomic-scale control over material architecture.

**Table 3:** Mechanistic comparison of water purification technologies

Purification mechanism	Conventional clay vessels	Carbon-based 2D materials (GO, rGO, MXenes)	Evolutionary advance
Size-exclusion sieving	Macro/micro-pores (0.1–10 µm): effective for bacteria/protozoa. Irregular, tortuous pathways limit flow and selectivity	Nano/angstrom-scale channels (<1–2 nm): Discriminate between hydrated ions (Na^+^*vs.* Mg^2+^). Creates ordered laminar flow in stacked membranes	Atomic precision: transition from stochastic porosity to engineered interlayer spacing for precise ion sieving. Pore size reduction 10^4^×; flux 100× higher
Adsorption	Electrostatic (cation exchange): Relies on inherent clay negative charge (CEC 10–150 meq/100g). Effective for cations but limited for neutrals/anions. Capacity: 10–50 mg g^−1^ Pb^2+^	Tunable surface chemistry: high-capacity, selective adsorption *via* coordination, electrostatic, π–π interactions. Capacity: 200–1500 mg g^−1^ Pb^2+^ (lab), 100–500 mg g^−1^ (real water)	Specificity & capacity: vastly increased surface area and chemically tunable active sites enable high-capacity selective removal. 10–50× higher capacity
Antimicrobial action	Silver ion leaching: broad-spectrum but uncontrolled release; potential bacterial resistance and environmental accumulation. Efficiency: 2–4 log reduction in 4–8 hours	Physical damage (nano-knives): sharp sheet edges disrupt membranes. Oxidative stress & photothermal killing: ROS generation or localised heating under light. Efficiency: 5-log in 2–4 hours (MXene), 4-log in 4–6 hours (GO)	Multi-modal & contact-based: moves beyond leaching biocides to direct physical and catalytic mechanisms, reducing environmental impact and resistance
Catalytic degradation	Not a typical traditional clay feature	Photocatalysis & electrocatalysis: activate PMS or generate hydroxyl radicals to mineralise persistent organics. Example: rGO-PMS degrades 90% bisphenol A in 30 min	Contaminant destruction: transforms filtration from containment to complete mineralisation

## 7. Application-oriented design of two-dimensional materials

Graphene oxide membranes serve as typical examples of this technology era, especially comprising stacked laminates of millions of individual graphene oxide sheets, creating well-defined two-dimensional nanochannel networks.^154–156^

### 7.1. The modern pore: tunable interlayer spacing

The fundamental filtration unit in graphene oxide membranes is the interlayer spacing (*d*-spacing), meticulously engineered voids created by precise stacking of thin atomic sheets. This *d*-spacing functions as an efficient molecular-scale pore equivalent with perfection and uniformity unattainable by traditional materials. Unlike fixed, irregular clay matrix pores, these nano-scale galleries are dynamic and exquisitely tunable. Water molecule presence itself acts as a tuning key; water molecules intercalate between sheets, gently propping them apart to specific heights. By carefully controlling hydration levels, researchers effectively set channel widths like adjusting molecular-scale calipers. Applied pressure offers real-time control, subtly compressing layers to narrow passages and selectively sieve smaller species. For permanent robust precision, covalent cross-linking stitches sheets together at predetermined distances, creating stable, rigid pore architectures, maintaining exact dimensions under demanding conditions.

The ultimate goal of tunability is molecular discrimination, designing membranes excluding specific ions while allowing others to pass. In theory, *d*-spacing can be engineered to block larger hydrated ions like Mg^2+^ while permitting smaller ones like Na^+^ to flow through. This selective rejection based on hydrated size represents filtration control pinnacles. Such engineering feats are unimaginable in clay matrices where pore sizes are statistically distributed and fixed by random mineral particle arrangements. GO membranes with *d*-spacing 0.9 nm demonstrate >95% rejection of Na^+^ (hydrated radius 0.36 nm) while allowing water permeance of 0.5–5 L m^−2^ h^−1^ bar^−1^, exceeding conventional polymeric membranes by 2–10 times. However, swelling increases *d*-spacing to 1.2–1.5 nm in water, reducing Na^+^ rejection to 60–70%. Cross-linking (*e.g.*, with ethylenediamine) stabilises spacing but reduces water permeability by 30–50%.^157–159^

### 7.2. Multifunctional surfaces: reactive barriers

Graphene oxide sheet surfaces function as dual-purpose platforms combining physical sieve and molecular scavenger roles. While a two-dimensional structure provides fundamental physical barriers, true power lies in dense chemical landscapes painted across surfaces. This landscape features high-density oxygen-containing functional groups (hydroxyls, epoxides, carboxyls). These groups function not as impurities but as versatile chemical toolkits capable of grabbing different contaminant types. High-density arrangements mean single sheets present immense interaction opportunities, maximizing cleaning capacity. Consequently, graphene oxide membranes can perform multiple purification tasks in integrated steps rather than requiring separate filter series.^160–162^

As water flows through nanochannels, heavy metal ions are captured through strong coordination bonds with oxygen groups. Simultaneously, polar organic molecules (certain pesticides, dyes) are adsorbed *via* electrostatic attractions or hydrogen bonding. This enables the material to tackle complex waste streams containing diverse pollutant cocktails simultaneously. The process is not sequential but concurrent; adsorption occurs alongside size-based separation, creating powerful synergistic effects. Contaminants small enough to pass through physical pores might still be captured and immobilized by chemical adsorption on pore walls. This multifunctionality dramatically departs from traditional adsorbents like activated carbon, offering primarily surface area without precise sieving benefits. In clay filters, physical and chemical processes are largely independent and limited; in graphene oxide, they are intrinsically linked and amplified.^163–165^

### 7.3. Enhanced flow: overcoming nano-scale limitations

One of the most captivating paradoxes in the above context is achieving remarkable flow rates despite almost impossibly small pores. Intuition suggests reducing pores to atomic scales should create immense resistance, grinding water flow to near halts. However, within material nanochannels, different realities emerge governed by unique nanoscale physics. The secret lies in the region of pristine graphene having a hydrophobic nature. Unlike rough, sticky conventional material internal surfaces, these carbon surfaces are atomically smooth and slightly repel water molecules. This combination of supreme smoothness and low chemical attraction means water molecules encounter minimal friction traversing channels. Instead of sticking and tumbling chaotically, water molecules organize into ordered chains or layers, sliding past surfaces surprisingly easily.

This phenomenon, often described as slip flow, allows water to zip through constricted nanochannels with minimal energy loss. The result is membranes defying classical expectations, offering both exceptional selectivity and high permeability. It's the difference between pushing water through long, rough, sand-filled tubes *versus* letting it glide down short, polished ice slides. Consequently, permeability, the water passage measure under given pressure, can surpass many conventional polymeric membranes.^166,167^ This is a critical advantage directly translating to lower filtration process energy requirements (reverse osmosis, nanofiltration). While traditional polymeric membranes often face selectivity, permeability trade-offs, graphene-based materials challenge this compromise. Hydrophobic graphene acts as molecular speedways while functionalized areas and precise pore sizes act as toll booths and filters. Enhanced flow means less pumping pressure is required to achieve the same purified water output, leading to significant energy savings.^168–170^ Flow is not just fast but exceptionally efficient, as minimal input energy overcomes internal friction.

### 7.4. Critical operational challenges

Transitioning graphene oxide (GO) membranes from bench-scale curiosities to viable water-treatment assets necessitates a fundamental paradigm shift, moving beyond the pursuit of pristine material perfection in idealized deionized-water settings toward an integrated engineering mindset that fully embraces real-world complexity. The following subsections converge on the four most pivotal, and frequently underplayed, hurdles: osmotic swelling, multicomponent fouling, manufacturing scalability, and operational performance drift under authentic feed streams. Critically, these are not independent variables; rather, they form a tightly interconnected network where a change in one invariably exacerbates or mitigates another. Collectively arguing that only through orchestrated, cross-disciplinary development, anchored by transparent pilot-scale validation, can GO membranes achieve the durability, selectivity, and economic feasibility demanded by municipal and industrial practitioners.

#### 7.4.1. Membrane swelling

Graphene oxide (GO) membranes are intrinsically hygroscopic owing to their abundant oxygen-containing functional groups (hydroxyl, epoxy, and carboxyl). Upon exposure to aqueous feed streams, water molecules readily intercalate between the stacked nanosheets, driving a progressive increase in the interlayer *d*-spacing, often expanding from 0.8 nm in the dry state to beyond 1.2 nm under fully hydrated conditions, depending on the degree of confinement and solution ionic strength. This enlarged gallery height severely compromises the primary size-exclusion and electrostatic rejection mechanisms, leading to a marked loss of selectivity for both monovalent and divalent ions. To counteract this inherent instability, several mitigation strategies have been proposed. Cross-linking agents (*e.g.*, ethylenediamine, glutaraldehyde, or borate ions) form covalent or ionic bridges between adjacent sheets, effectively restraining interlayer expansion *via* mechanical tethering; however, the introduced steric hindrance and reduced free volume typically lower water permeance by 30–50%, while often rendering the membrane more brittle and prone to micro-cracking under pressure. Partial thermal or chemical reduction decreases the density of hydrophilic groups to limit water uptake, but this comes at the cost of impaired dispersibility during fabrication and a reduction in negative surface charge, which diminishes electrostatic repulsion for anionic contaminants. Intercalating monovalent (K^+^, Na^+^) or divalent (Ca^2+^, Mg^2+^) cations screens the negative basal-plane charges and compresses the *d*-spacing *via* electrostatic bridging, yet this approach is susceptible to cation leaching under dynamic cross-flow conditions and in low-ionic-strength feeds. Hydrophobic top-coatings (*e.g.*, polydimethylsiloxane or perfluorinated layers) offer a barrier against bulk water penetration, but they introduce additional hydraulic resistance, may delaminate under high shear stresses, and often reduce the effective surface area for permeation. Each intervention thus presents a compromise that must be carefully weighed against the targeted separation performance and operational durability.^171–174^

#### 7.4.2. Fouling

In realistic wastewater matrices, the membrane surface rapidly accumulates a complex and dynamic foulant layer composed of humic substances, polysaccharides, proteins, and extracellular polymeric substances (EPS) from microbial consortia, alongside inorganic colloidal particles and precipitating scalants. This combined organic–bio–inorganic fouling layer induces a precipitous flux decline of 30–60% within the first 24 to 72 hours of continuous operation, driven primarily by concentration polarization, subsequent cake/gel layer formation, and pore-blocking phenomena that exacerbate hydraulic resistance. While surface modifications using hydrophilic polymers (*e.g.*, PEG chains or zwitterionic sulfobetaine polymers) enhance fouling resistance through steric repulsion and robust surface hydration layers, their long-term stability against hydrolysis, enzymatic degradation, and oxidative attack under real effluent conditions remains questionable, with performance often degrading within weeks. Photocatalytic nanoparticles such as TiO_2_ offer a self-cleaning avenue *via* the generation of reactive oxygen species (ROS) under UV irradiation, yet UV penetration is severely attenuated in turbid, high-turbidity wastewaters, and the required lamp infrastructure substantially escalates both capital and operational energy costs. Conventional physical backwashing and chemical cleaning (using NaOCl, citric acid, or caustic soda) are effective for short-term flux recovery, but they impose cyclical mechanical stresses and chemical attacks that progressively oxidize the GO basal planes, strip essential oxygen functional groups, induce edge-initiated delamination, and weaken the adhesion between the selective layer and the porous support. Consequently, the membrane's effective operational lifespan can be shortened by 40–60% over repeated cleaning cycles compared to pristine, unexposed specimens, necessitating the development of gentler, *in situ* cleaning protocols and foulant-release surface topographies.^175–179^

#### 7.4.3. Scalability

Transitioning from laboratory-scale (cm^2^) coupons to industrially relevant membrane modules (m^2^ scale) represents a formidable manufacturing bottleneck that remains insufficiently addressed. Current solution-based assembly methods, including vacuum filtration, spray-coating, slot-die coating, meniscus-guided deposition, and Langmuir–Blodgett transfer, each face intrinsic trade-offs between deposition speed and structural precision. Achieving pinhole-free, continuous films with sub-nanometer thickness uniformity over meter-length scales is persistently thwarted by the polydisperse lateral size distribution of GO flakes, edge-induced stress cracking during drying, and the profound influence of underlying support roughness (*e.g.*, polymeric ultrafiltration substrates) on film continuity and defect density. Roll-to-roll (R2R) processing has been championed as the most viable pathway for continuous, high-throughput production; nevertheless, the high web speeds (typically 1–5 m min^−1^) and associated shear forces involved in such processes tend to disturb the preferred lamellar ordering and induce macroscopic stripe defects. As a result, R2R-fabricated membranes generally exhibit water permeance and rejection metrics that are 30–50% lower than those achieved *via* static, laboratory-scale casting, undermining the economic rationale for scale-up. Furthermore, the high raw-material cost of synthesizing high-purity GO (involving harsh oxidizing agents, temperature control, and lengthy purification/dialysis steps), coupled with substantial solvent management and waste-disposal challenges at production scale, imposes significant economic pressure. Realizing industrial viability will therefore require parallel advances in low-defect, large-flake GO precursor synthesis, continuous in-line quality-control metrology (*e.g.*, spectral reflectance or eddy-current thickness monitoring), and the adoption of hybrid casting techniques that combine the speed of R2R with the precision of shear-aligned deposition.^180–183^

#### 7.4.4. Real-world performance

Laboratory evaluations conducted using deionised (DI) water spiked with model salts are fundamentally non-predictive of field performance, as they systematically neglect the synergistic effects of competing ionic species, fluctuating pH, natural organic matter (NOM), colloidal turbidity, and variable hydrodynamic shear that are ubiquitous in municipal and industrial effluents. Under authentic municipal wastewater conditions, the baseline permeate flux typically plummets to 40–60% of its initial DI-water value within the first operational day, while the apparent rejection of mixed ionic species collapses from >95% in ideal settings to a starkly diminished 50–70%. This drastic deterioration is not merely a flux effect; it stems from a confluence of membrane compaction under hydraulic pressure, foulant-induced pore blockage, disruption of charge-exclusion mechanisms by high-ionic-strength backgrounds, and the weakening of interlayer hydrogen-bonding networks in the presence of chaotropic ions. Crucially, long-term stability over weeks or months of continuous operation remains almost entirely uncharacterized, leaving critical questions about chemical aging, structural fatigue, and irreversible fouling largely unanswered. To date, only a handful of pilot-scale investigations have been documented in the peer-reviewed literature; notably, several reports typically employ membrane areas of less than 1 m^2^ and operate for durations under 200 hours, making their extrapolation to full-scale plants highly speculative. It is strongly advocated for a systematic, meta-analytical review of existing pilot data to establish baseline performance decay models and identify critical operating windows; however, the current corpus is too nascent, heterogeneous in testing protocols, and fragmented in reporting metrics to yield statistically robust or universally applicable conclusions. Standardised testing guidelines, incorporating realistic cross-flow velocities, intermittent backwashing schedules, feed pre-treatment variability, and temperature-controlled conditions—are urgently needed to enable meaningful cross-comparison between different membrane formulations and operating regimes.^184–188^

#### 7.4.5. Path forward

A viable trajectory toward commercialization cannot rest solely on bespoke material chemistries; instead, it demands a holistic, tiered engineering framework that integrates material innovation, process design, and rigorous field validation from the outset. Material-level advances, such as mixed-matrix GO architectures incorporating nano-fillers, covalent interlayer tethering using rigid molecular linkers, and bio-inspired adhesive binders to enhance mechanical cohesion, must be co-developed with robust process engineering solutions. These include optimized feed pre-treatment trains (*e.g.*, coagulation/flocculation, ozonation, or granular activated carbon filtration) to substantially reduce the upstream foulant load, as well as advanced module designs incorporating feed spacers with turbulent-promoting geometries to mitigate concentration polarization and enhance mass transfer. Pilot- and demonstration-scale plants (treating >10 m^3^ per day) must become a routine, mandatory milestone in the research-and-development lifecycle, moving well beyond proof-of-concept benchtop experiments. The integration of real-time, non-destructive monitoring tools, such as electrical impedance spectroscopy, ultrasonic reflectometry, or optical coherence tomography, could provide critical feedback for adaptive cleaning schedules and predictive maintenance, thereby extending membrane lifespan. Ultimately, only through a balanced and sustained investment in scalable manufacturing, durable antifouling surface engineering, and long-term, real-world field trials under diverse feedwater chemistries can graphene oxide membranes transition from a promising laboratory curiosity to a reliable, cost-competitive, and commercially deployable technology for next-generation water treatment and resource recovery.^189–193^

## 8. Critical analysis: performance, limitations, and practical challenges

**Fig. 8:**
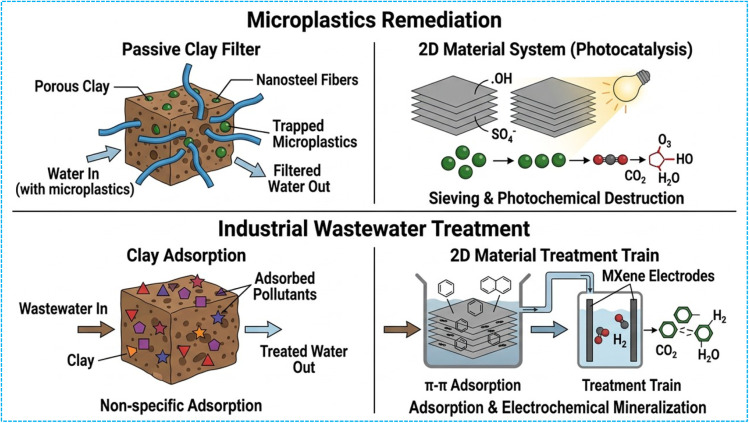
Multi-modal attack on microplastics and industrial waste.

While two-dimensional materials demonstrate extraordinary capabilities, critical evaluation reveals significant challenges requiring resolution before widespread implementation.^194,195^ This section systematically analyzes performance metrics, identifies limitations, and addresses practical implementation barriers (Fig. 8).

### 8.1. Performance comparison across contaminant classes

#### 8.1.1. Heavy metals

As given in Table 4, graphene oxide exhibits exceptional adsorption capacities (200–1500 mg g^−1^ depending on metal and conditions), significantly surpassing activated carbon (typically 10–200 mg g^−1^) and clay minerals (1–50 mg g^−1^).^196^ However, performance depends strongly on pH, competing ions, and organic matter. In complex wastewater matrices, graphene oxide capacity can decrease by 30–70% compared to single-contaminant laboratory conditions. MXenes show particular promise for redox-active metals like chromium, combining adsorption with reduction to less toxic species, but require precise pH control (typically pH 2–4) that may necessitate pre-treatment.^197,198^ The high lab capacities are rarely achieved in practice; for example, GO Pb^2+^ capacity drops from 842 mg L^−1^ (DI water) to 250 mg L^−1^ in the presence of 100 mg L^−1^ humic acid and competing cations.

**Table 4:** Heavy metal adsorption capacity comparison

Material	Target ion	Lab capacity (mg g^−1^)	Real-water capacity (mg g^−1^)	pH optimum	Regeneration (%)	Key mechanism	Limitation
GO	Pb^2+^	850–1200	200–500	5–6	Acid wash (80%)	Coordination –COOH	Competing ions, NOM
GO	Cd^2+^	150–300	50–100	6–7	Acid wash (70%)	Electrostatic + coordination	Ca^2+^/Mg^2+^ interference
MXene	Cr(vi)	200–400	100–200	2–3	Alkaline wash (85%)	Reduction + adsorption	pH adjustment needed
Clay	Pb^2+^	10–50	5–20	6–7	Limited	Cation exchange	Low capacity

#### 8.1.2. Organic pollutants

Reduced graphene oxide and functionalized derivatives achieve rapid degradation of persistent organics through advanced oxidation processes. For example, reduced graphene oxide-activated peroxymonosulfate (PMS) systems degrade >90% of bisphenol A within 30 minutes, compared to 2–4 hours for conventional Fenton processes.^199,200^ However, catalyst leaching, byproduct formation, and high oxidant requirements present practical challenges. Photocatalytic graphene oxide–titanium dioxide composites show enhanced visible-light activity but suffer from electron–hole recombination and limited light penetration in turbid waters. rGO-PMS systems achieve 95% bisphenol A degradation in 20 minutes *versus* 60 minutes for activated carbon-PMS. However, catalyst leaching (*e.g.*, metal ions if doped) and by-product formation (*e.g.*, bromate in bromide-containing waters) require thorough evaluation for environmental safety.^201,202^

#### 8.1.3. Pathogens

MXenes demonstrate superior antimicrobial activity (4–5 log reduction in 2–4 hours) compared to silver nanoparticles (3–4 log reduction in 4–6 hours) and conventional disinfectants.^203,204^ The multi-modal mechanism (physical disruption, oxidative stress, photothermal effects) reduces resistance development potential. However, nanomaterial recovery, residual toxicity, and disinfection byproduct formation require thorough investigation. Long-term exposure effects on beneficial microorganisms and ecosystem impacts remain largely unknown.^205^ Chlorine still outperforms in speed (>6-log in 30 min) but produces harmful by-products. 2D materials may find niches in point-of-use or decentralised systems.^206,207^

#### 8.1.4. Microplastics and emerging contaminants

Graphene oxide membranes with controlled *d*-spacing achieve >99% removal of nanoplastics (100–500 nm) through combined sieving and adsorption. This significantly outperforms conventional ultrafiltration membranes that typically remove only larger microplastics (>0.1 µm) with 70–90% efficiency.^208,209^ However, membrane fouling by organic matter and biofilm formation reduces performance over time, requiring periodic cleaning and potentially limiting operational lifetimes. GO membranes with *d*-spacing 1.2 nm achieve >99% removal of 100 nm polystyrene nanoparticles; photocatalytic GO-TiO_2_ composites can further degrade captured microplastics. Nevertheless, flow rates remain a challenge, with typical permeance of 1–3 L m^−2^ h^−1^ bar^−1^, often insufficient for large-scale treatment.^210–212^ Regeneration efficiency rarely exceeds 80% over multiple cycles, indicating gradual performance loss, a major limitation for practical application.^213,214^

### 8.2. Scalability and manufacturing challenges

Current two-dimensional material synthesis methods face significant scalability hurdles. The Hummers' method for graphene oxide production generates substantial hazardous waste (acid, oxidant residues), raising environmental concerns.^215,216^ MXene synthesis typically requires hazardous hydrofluoric acid or fluoride salts for etching, posing safety and disposal challenges. While alternative methods (electrochemical exfoliation, microwave-assisted synthesis) show promise, they often yield materials with inconsistent properties and lower performance.^217,218^

Large-area membrane fabrication presents additional challenges. Even minor defects (0.01–0.1% area) can significantly compromise selectivity by providing bypass pathways for contaminants. Current fabrication techniques (vacuum filtration, spin coating, layer-by-layer assembly) are difficult to scale beyond laboratory dimensions (typically <100 cm^2^). Continuous roll-to-roll manufacturing, essential for industrial adoption, remains largely undeveloped for two-dimensional material membranes.^219,220^

Cost represents another critical barrier. Current graphene oxide production costs ($100–500 per g^−1^) and MXene costs (>$1000 per g^−1^) prohibit large-scale water treatment applications.^221,222^ While performance advantages may justify premium costs for specific high-value applications (pharmaceutical removal, precious metal recovery), widespread adoption requires at least 10–100 times cost reduction through improved synthesis methods and scaled production.

### 8.3. Stability and fouling issues

Graphene oxide membranes swell in aqueous environments, increasing *d*-spacing and reducing selectivity over time. Cross-linking improves stability but typically decreases permeability by 20–50%. In saline or high-ionic-strength waters, graphene oxide membranes can undergo structural rearrangement or even disintegration. MXenes face oxidation challenges, particularly in aerated water, gradually losing conductivity and functionality.^223,224^

Fouling remains a persistent challenge. Organic matter, biological growth, and particulate accumulation reduce water flux by 30–70% within days to weeks, depending on water quality. While two-dimensional materials possess smooth surfaces and tunable chemistry offer fouling resistance advantages compared to conventional membranes, practical anti-fouling strategies (surface modification, pulsatile operation, chemical cleaning) add complexity and cost. Regeneration efficiency rarely exceeds 80% over multiple cycles, indicating gradual performance loss that ultimately limits operational lifespan.^225–227^

### 8.4. Environmental and health implications

Nanomaterial release during operation and membrane disposal pose potential ecological risks. Graphene oxide shows moderate aquatic toxicity at concentrations >10 mg L^−1^, affecting microbial communities and aquatic organisms.^228,229^ Specific acute toxicity data shows that LC_50_ for *Daphnia magna* as 10–50 mg L^−1^, for zebrafish embryos 50–100 mg L^−1^, for algae 20–50 mg L^−1^. Chronic sublethal effects occur at lower concentrations (1–10 mg L^−1^) and include oxidative stress, reproductive impairment, and growth inhibition.^230^

MXene toxicity remains poorly characterized, but preliminary studies suggest similar concerns. Lifecycle analysis, essential for sustainable technology assessment, is notably absent from most studies.^231,232^ The nano-enabling of water treatment must consider not only immediate purification benefits but also long-term environmental implications. Material persistence, transformation products, and ecological impacts across food chains require comprehensive investigation. Regulatory frameworks for nanomaterial use in water treatment remain underdeveloped, creating uncertainty for technology adoption and commercialization. Nanomaterial release during operation and membrane disposal pose ecological risks. GO shows moderate aquatic toxicity at >10 mg L^−1^, while MXene toxicity remains poorly characterized. Lifecycle analysis is notably absent from most studies, a critical omission for sustainable technology assessment.^233–235^

### 8.5. System integration and operational considerations

Most research examines isolated materials under idealized conditions (single contaminants, pure water matrices, optimized pH).^236–238^ Real-world implementation requires integrated systems addressing multiple contaminants simultaneously, variable water quality, and fluctuating operational parameters. Pretreatment requirements, energy consumption, maintenance protocols, and waste management must be considered holistically. Pilot-scale studies (1–100 m^3^ per day capacity) are essential for validating laboratory findings and identifying scale-up challenges.^239,240^ Few such studies exist for two-dimensional material water treatment systems, representing a critical knowledge gap. Techno-economic analysis comparing two-dimensional material systems with conventional alternatives across different applications and scales is needed to guide development priorities and investment decisions.^241–243^

### 8.6. Economic viability analysis

Table 5 demonstrates that at current prices, GO and MXenes are orders of magnitude more expensive than activated carbon for contaminant removal. High-value applications (*e.g.*, precious metal recovery, pharmaceutical removal from hospital wastewater) may justify the cost, but municipal water treatment is economically infeasible without drastic cost reduction. The pathways to gain cost reduction include large-scale production from graphite, using waste biomass as a precursor, electrochemical exfoliation to replace the Hummers' method, and recycling and reuse of nanomaterials.^241–243^

**Table 5:** Economic viability comparison

Material	Cost ($ per g)	Pb^2+^ capacity (mg g^−1^)	Cost per mg Pb removed ($ per mg)	Relative cost *vs.* AC
Activated carbon	0.001–0.005	100	0.00001–0.00005	1×
GO (lab)	100–500	842	0.12–0.59	10 000–50 000×
GO (bulk projected)	10–50	842	0.012–0.059	1000–5000×
MXene (lab)	>1000	200–400	2.50–5.00	>1 000 00×

## 9. Future perspectives: from laboratory innovation to sustainable implementation

The transition from promising laboratory results to practical water treatment solutions requires coordinated advancement across multiple fronts. This section identifies key research directions and implementation strategies essential for realizing the potential of two-dimensional materials in addressing global water challenges.^244^

### 9.1. Advanced material design and engineering

Future research must pivot from repurposing existing materials to designing them specifically for water treatment, with four promising directions: defect engineering to precisely introduce vacancies, dopants, or functional groups that optimize adsorption, catalysis, and transport without compromising structural integrity; Janus membranes featuring asymmetric surface chemistries or pore architectures to enable multi-stage purification in a single unit and mitigate fouling *via* directional flow; stimuli-responsive materials that adapt pore size, charge, or wettability to fluctuating water quality through external triggers; and hybrid composites that integrate two-dimensional materials with conventional supports to synergistically combine strengths while offsetting individual weaknesses.^245–247^ Concrete five-year milestones include reducing graphene oxide production costs below $10 per gram, achieving membrane lifetimes exceeding one year in real-world conditions, maintaining over 90% regeneration efficiency across 20 cycles, and demonstrating pilot-scale validation at capacities above 100 litres per day.

### 9.2. Sustainable synthesis and manufacturing

Practical implementation demands production methods that are both environmentally benign and industrially scalable, achievable through green synthesis routes, such as electrochemical exfoliation, microwave-assisted techniques, and biological reduction, to minimise reliance on hazardous chemicals and energy consumption, alongside continuous manufacturing innovations like roll-to-roll processing and spray deposition for large-area, consistent membrane fabrication. Complementing these advances are standardised characterisation protocols and performance metrics to ensure batch-to-batch reliability and enable meaningful comparative evaluation, while embedded circular economy principles, designing materials and processes for recovery, regeneration, and reuse, ensure resource efficiency and waste minimisation across the entire technology lifecycle.^248–251^

### 9.3. System integration and performance validation

Transitioning from material development to fully functional water-treatment systems demands a holistic, systems-level approach. This progression begins with extended pilot-scale testing using authentic water matrices, municipal wastewater, industrial effluents, and contaminated groundwater, to uncover operational challenges and refine system designs under realistic conditions.^252–257^ Crucially, evaluation must shift from single-pollutant targets to multi-component treatment, addressing complex contaminant mixtures while accounting for competitive effects and synergistic interactions to optimise the entire treatment train. Practical deployment further relies on hybrid system designs that intelligently integrate two-dimensional material components with established conventional processes—such as coagulation, biological treatment, and disinfection—thereby creating robust, synergistic trains that harness the strengths of each technology. Complementing these technical strides, rigorous techno-economic analyses are indispensable, providing comprehensive cost-benefit comparisons against conventional alternatives across varied applications, scales, and geographical settings to confirm economic viability and guide strategic implementation.

### 9.4. Environmental safety and regulatory development

Responsible technology development rests on four interconnected pillars: systematic risk assessment, tracking the environmental fate, transport, transformation, and multi-trophic ecological effects of materials through controlled laboratory studies, mesocosm experiments, and predictive modelling; comprehensive lifecycle analysis to quantify environmental burdens from raw material extraction and synthesis through to use and end-of-life management, thereby identifying sustainability “hot spots” to guide iterative design; intrinsic safe-by-design approaches that embed safety considerations into material and system engineering from the very first development stages rather than retrofitting them later; and proactive collaboration with policymakers, industry stakeholders, and public health experts to forge robust, science-based regulatory frameworks, standards, and guidelines for nanomaterial application in water treatment.^258,259^ Given these imperatives, initial market entry will logically target high-value niches where performance and economic drivers align, namely, pharmaceutical removal from hospital wastewater (fueled by regulatory pressure and premium pricing), precious metal recovery from industrial effluents (where recovered material value offsets treatment costs), and point-of-use devices for emergency or decentralised applications (where reliability and safety decisively outweigh cost constraints).

### 9.5. Knowledge translation and capacity building

**Fig. 9:**
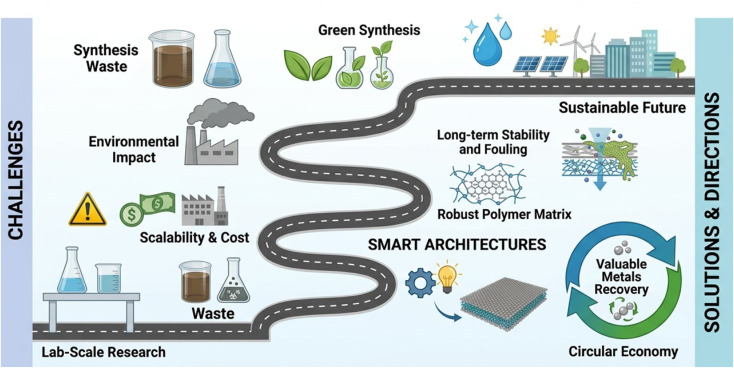
The path forward for 2D material-based water purification.

Fostering partnerships between materials scientists, environmental engineers, chemists, biologists, toxicologists, economists, and social scientists to address complex challenges holistically is all-important. Moreover, developing effective pathways for translating academic research into commercial products through industry partnerships, startup incubation, and supportive policies highlights the translation phenomenon of such fields. The path forward requires a multi-pronged strategy, as illustrated in Fig. 9.

## 10. Conclusion

This review has traced the conceptual and technological evolution from ancient clay-based water purification systems to contemporary two-dimensional nanomaterials, establishing continuity in fundamental mechanisms while highlighting transformative advances in precision, efficiency, and functionality. Carbon-based two-dimensional materials, graphene, graphene oxide, reduced graphene oxide, and MXenes, offer exceptional properties enabling unprecedented control over water decontamination processes at atomic and molecular scales. The progression from stochastic clay pore networks to engineered angstrom-scale channels represents not merely incremental improvement but a paradigm shift in separation science. Two-dimensional materials execute historical purification principles, sieving, adsorption, and disinfection, with precision and effectiveness unimaginable in traditional systems while introducing entirely new capabilities such as catalytic degradation. This evolutionary advance embodies the refinement and augmentation of core concepts rather than their replacement, connecting ancient material wisdom with modern scientific innovation.

Despite remarkable laboratory demonstrations, significant challenges remain in scalability, stability, cost, environmental safety, and practical implementation. Addressing these challenges requires coordinated advancement across material design, sustainable synthesis, system integration, risk assessment, and regulatory development. Future progress depends on balancing fundamental research with applied development, laboratory innovation with field validation, and technological potential with environmental responsibility. The historical continuity from clay to graphene reminds us that materials innovation builds upon accumulated knowledge while introducing transformative capabilities. As we advance these atomic-scale technologies, we must remain mindful of both their extraordinary potential and their practical limitations, guiding development toward sustainable, equitable water treatment solutions that address pressing global needs while preserving environmental integrity for future generations.

## Author contributions

Arwa A. Makki and Dina Hajjar: conceptualization, formal analysis, investigation, methodology, writing – original draft; Hatem M. Altass: conceptualization, formal analysis, funding acquisition, investigation; A. Timoumi: writing – review and editing, validation; Jan Mohammad Mir and Bashir Ahmad Malik: conceptualization, validation, investigation, methodology, project administration, resources, supervision, validation, writing – review and editing; Saleh A. Ahmed: conceptualization, formal analysis, funding acquisition, investigation, methodology, project administration, resources, supervision.

## Conflicts of interest

No potential conflict of interest was reported by the authors.

## Funding

This research work was funded by Umm Al-Qura University, Saudi Arabia under grant number: 26UQU4340244GSSR04.

## Data Availability

No primary research results, software, or code have been included in this review article, and no new data were generated or analysed as part of this work.
